# Complete chloroplast genome of *Sphagnum subsecundum* Nees from Korea

**DOI:** 10.1080/23802359.2021.1999186

**Published:** 2021-11-22

**Authors:** Wonhee Kim, Seah Ryu, Ji-Hee Song, Sunhee Sim

**Affiliations:** Plant Resources Division, National Institute of Biological Resources, Incheon, Republic of Korea

**Keywords:** Chloroplast genome, Jeju Island, *Sphagnum subsecundum*

## Abstract

We determined the complete chloroplast genome sequence of the peatmoss *Sphagnum subsecundum* Nees from Mt. Halla in Korea. The total size of the chloroplast genome was 140,136 bp, and it consisted of a large single-copy region (LSC) of 98,064 bp, a small single-copy region (SSC) of 21,388 bp, and two copies of inverted repeat (IRa and IRb) regions of 10,342 bp each. The genome encoded a set of 130 genes, comprising 85 protein-coding genes, 37 tRNA genes, and 8 rRNA genes. This species formed a monophyletic clade with *S. orientale* and *S. lenese* as the result of ML phylogenetic analysis based on whole gemone sequence of 15 species including one outgroup species.

The class Sphagnopsida consists of two families, the Ambuchananiaceae and Sphagnaceae. The Ambuchananiaceae is endemic to Tasmania, while the Sphagnaceae has a worldwide distribution. The Sphagnaceae consists of a single genus *Sphagnum* and 285 species (McQueen and Andrus 2007). Recently, this genus was divided into five subgenera based on organellar phylogenomic analyses (Shaw et al. 2016). On the Korean Peninsula, 22–29 species have been reported (Choe and Choi 1980; Hwang 1991; Kim et al. 2020). One of these species, *S. subsecundum* grows on an alpine wetland of Mt. Halla on Jeju Island. Jeju Island (Jeju-do or ‘Quelpart’) is the largest island in South Korea. The island is about 240 km in circumference and Mt. Halla, a shield volcano, is located in the center of the island, and also the highest mountain in South Korea. In Korea, the distribution of *S. subsecundum* is restricted to Jeju Island, but has been reported across most continents, primarily in boreal regions (Gao et al. 1999; McQeen and Andrus 2007). This study analyzed the chloroplast genome of *S. subsecundum* collected on the Seonjakjiwat Plain on Mt. Halla, which is an alpine wetland meadow.

Fresh samples were collected from Mt. Halla, Jeju Island in South Korea. A voucher specimen (WKim 2017-05-17, W. Kim, spitz8823@korea.kr) was deposited in the herbarium of the National Institute of Biological Resources (KB, http://www.nibr.go.kr). The genomic DNA was isolated from the fresh leaves using the DNeasy Plant Mini Kit (Qiagen) and Illumina paired-end (PE) library was constructed with an average 670 bp insert size, following the manufacturer’s instructions. The library was sequenced using an Illumina MiSeq platform by LabGenomics (www.labgenomics.co.kr, Seongnam, Korea). High-quality paired-end (PE) reads of about 1.4 Gb were employed for a *de novo* assembly using the default settings in CLC genome assembler (v. 4.21, CLC Inc., Aarhus, Denmark). A detailed procedure was described in the previous study (Lee et al. 2018). Among the assembled contig sets, putative chloroplast contigs were selected and merged into a single draft sequence compared with the reference sequence of *S. palustre* (GenBank accession no. KC784957). The draft sequence was manually corrected and gap-filled by iterative PE read mapping. The final complete chloroplast genome sequence was annotated using GeSeq (https://chlorobox.mpimp-golm.mpg.de/geseq-app.html) and was manually curated using Artemis annotation tool with NCBI BLASTN searches (Rutherford et al. 2000). The annotation was performed based on *S. orientale* (KU725447) and *S. palustre* (KU726621) using Geneious Prime 11.0.4 + 11 (Biomatters Ltd., Auckland, New Zealand). The complete chloroplast genome of *S. subsecundum* is a circular chromosome with a length of 140,136 bp. The genome has a typical quadripartite structure, and consists of two copies of inverted repeats (IRa and IRb: 10,342 bp each) separated by two regions: the large single-copy region (LSC: 98,064 bp) and the small single-copy region (SSC: 21,388 bp). The total GC content is 36.8%, and that of the LSC, SSC, and each IR was 35, 34.1, and 48.3%, respectively. The chloroplast genome encodes a set of 130 genes, comprising 85 protein-coding, 37 tRNA, and 8 rRNA genes. Chloroplast genome content and organization in *S. subsecundum* was mostly similar to previously published species (Shaw et al. 2016).Like the other species of *Sphagnum*, the *pet*N and *cys*T genes are recognized here as a miscellaneous gene and a pseudogene in *Sphagnum,* respectively, which is annotated by the previous study (Shaw et al. 2016). However, the two genes are not present in this study. The subgeneric relationships within *Sphagnum* are supported by phylogenetic analysis of organelle genomes (Shaw et al. 2016). However, *S. subsecumdum*, the type species of the subgenus *Subsecunda,* is not included in the analyses. As the analysis shows, three principle genetic variations are revealed. First, this species has the CAS stop codon, but UAG in the *ccs*A gene. This stop codon is also found in *S. orientale* (KU725447). As a second genetic difference, the *atp*F gene is identified. Usually, the *atp*F gene contains two CDS and one intron. This intron region length is 675 ∼ 692 bp, and there are interspecific variations. In the case of *S. subsecundum*, the intron is 692 bp in length, including 16 bp repeat region. Finally, this species differs from other species of *Subsecunda* in having a sequence deletion in the intergenic spacer region on *rps*4-*trn*T. This region is known as a plant DNA barcording region, and this study supports that this species has significant sequence deleted regions which are 20 base pairs in length. To examine the phylogenetic history of *S. subsecundum*, the chloroplast genome sequences of *S. subsecundum* and 13 species in *Sphagnum*, and one outgroup species were aligned by MAFFT version 7.45 (Katoh and Standley 2013), with the combined rapid bootstrap analysis (1000 replicates) and search for best-scoring ML tree (the ‘-f a – x 1′ option). The GTR GAMMA model was used for the maximum likelihood analysis. The phylogenetic analysis fully resolved *S. subsecundum* in clade with *S. orientale* and *S. lenense* (Figure 1). These phylogenetic results are similar to the organellar phylogenetic findings reported by Shaw et al. (2016).

**Figure 1.**
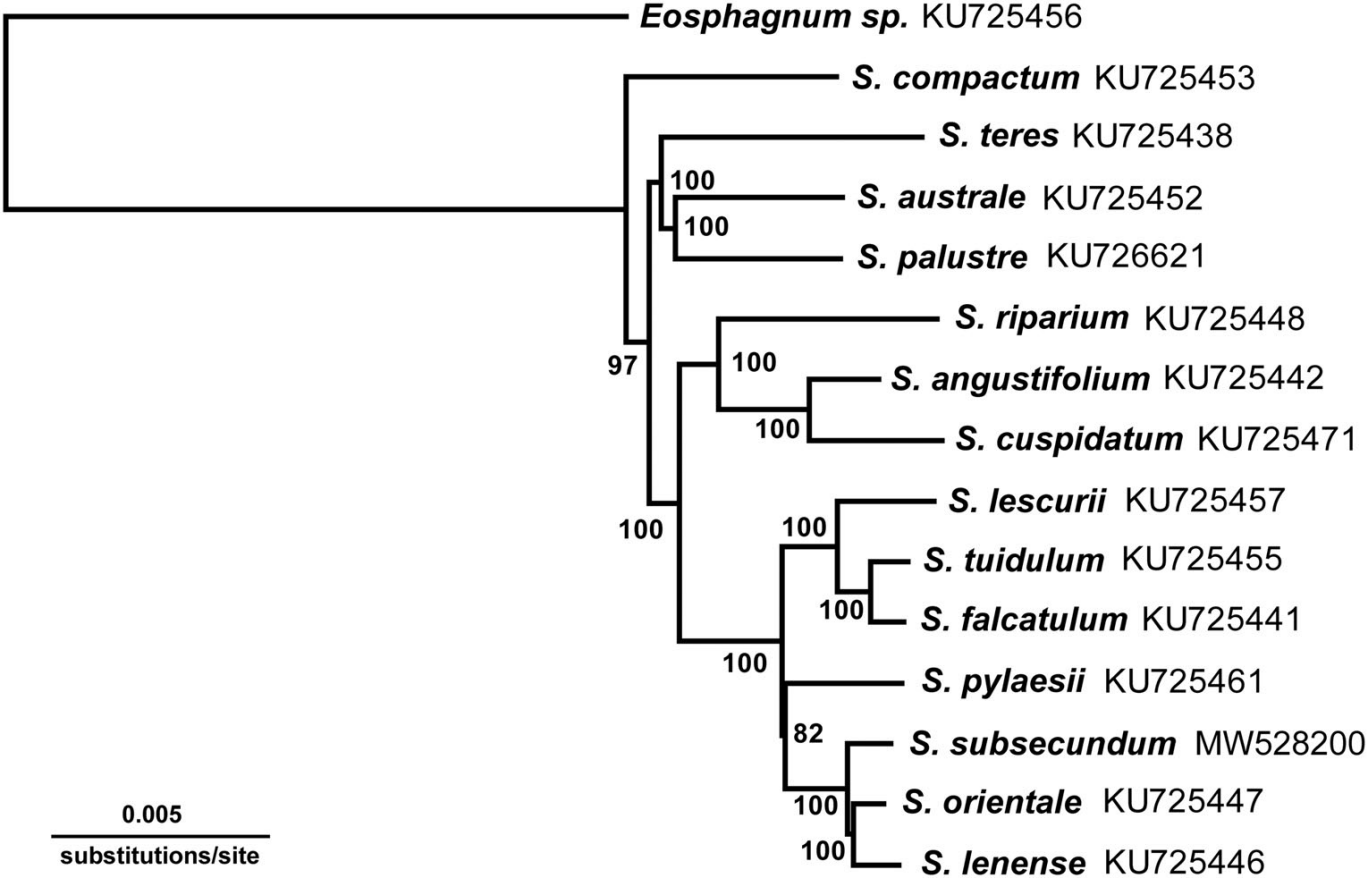
Phylogenetic tree inferred from maximum likelihood (RAxML v.8.2.11), based on complete chloroplast genome sequences of Sphagnum using GTR GAMMA model. Bootstrap support values (>50%) are indicated at each node.

## Data Availability

The data that support the findings of this study are openly available in the US National Center for Biotechnology Information (NCBI) database at http://www.ncbi.nlm.nih.gov/, reference number: MW528200. The associated BioProject, SRA, and Bio-Sample numbers are PRJNA728924, SRR14495356, and SAMN19107575, respectively.

## References

[CIT0001] Choe DM, Choi HH. 1980. A list of bryophytes of Korea. J Science Educ. 12:27–55.

[CIT0002] Gao C, Crosby MR, He S. 1999. Sphagnaceae-Leucobryaceae. 1: viii + 273. In: Moss flora of China. Beijing, New York, and St. Louis: Science Press & Missouri Botanical Garden.

[CIT0003] Hwang HJ. 1991. JosenSporic plants. Vol. 9, Bryophytes 2. Pyeongyan (North Korea): Science Encyclopedia Press; p. 391.

[CIT0005] Katoh K, Standley DM. 2013. MAFFT multiple sequence alignment software version 7: improvements in performance and usability. Mol Biol Evol. 30(4):772–780. 23329690 10.1093/molbev/mst010 PMC3603318

[CIT0006] Kim W, Higuchi M, Yamaguchi T. 2020. An updated list of mosses of Korea. J Species Res. 9(4):377–412.

[CIT0007] Lee HO, Choi JW, Baek JH, Oh JH, Lee SC, Kim CK. 2018. Assembly of the mitochondrial genome in the Campanulaceae family using Illumina low-coverage sequencing. Genes. 9(8):383. 10.3390/genes9080383 PMC6116063 30061537

[CIT0008] McQueen CB, Richard EA. 2007. Sphagnaceae. In: Flora of North America. Vol. 27. New York: Oxford University Press; pp. 45, 78–84.

[CIT0009] Rutherford K, Parkhill J, Crook J, Horsnell T, Rice P, Rajandream MA, Barrell B. 2000. Artemis: sequence visualization and annotation. Bioinformatics. 16(10):944–945. 11120685 10.1093/bioinformatics/16.10.944

[CIT0010] Shaw JA, Devos N, Liu Y, Cox CJ, Goffinet B, Flatberg KI, Shaw B. 2016. Organellar phylogenomics of an emerging model system: Sphagnum (peatmoss). Ann Bot. 118(2):185–196. 27268484 10.1093/aob/mcw086 PMC4970357

